# Association of delayed enteral nutrition with hospital-acquired pneumonia and 30-day mortality following craniotomy for severe traumatic brain injury: a retrospective cohort study

**DOI:** 10.3389/fnut.2026.1911672

**Published:** 2026-10-01

**Authors:** Zhiqiang Kang, Guodong Zhang, Wenjing Guo, Meng Liu, Dapeng Zhang, Fei Shang, Yang Wang

**Affiliations:** Department of Neurosurgery, Xinxiang Central Hospital, Xinxiang, China

**Keywords:** associated factors, craniotomy, enteral nutrition, hospital-acquired pneumonia (HAP), traumatic brain injury (TBI)

## Abstract

**Background:**

Hospital-acquired pneumonia (HAP) is a serious complication after craniotomy for severe traumatic brain injury (TBI), but evidence on modifiable factors, especially delayed enteral nutrition, remains limited.

**Objective:**

To evaluate factors associated with HAP after severe TBI surgery, focusing on enteral nutrition timing and the association between HAP and 30-day mortality.

**Methods:**

This retrospective cohort study included 600 adult patients undergoing craniotomy for severe TBI who survived >48 h. HAP was diagnosed according to CDC/NHSN criteria. Multivariable logistic regression identified factors associated with HAP, and a fully adjusted model assessed the HAP–mortality association. Model performance was assessed by the AUC and Hosmer–Lemeshow test. Sensitivity analyses included forced inclusion of tracheostomy and comparison of the primary model (pre-HAP ventilation duration) with the total ventilation model. A 48-h landmark analysis was also performed. The primary outcome was postoperative HAP; secondary outcomes were ICU stay and 30-day mortality.

**Results:**

Postoperative HAP incidence was 11.5%. After adjustment, delayed enteral nutrition (≥48 h) was independently associated with HAP (OR = 4.46, 95% CI: 2.52–8.19, *p* < 0.001). Other associated factors included age, admission GCS, mechanical ventilation duration, and serum albumin. The model showed good discrimination (AUC = 0.795) and calibration (Hosmer–Lemeshow *p* = 0.829). In the time-dependent Cox model, HAP was associated with higher 30-day mortality (HR = 4.31, 95% CI: 1.52–12.25, *p* = 0.006). The primary model and total ventilation model yielded nearly identical results. In the landmark analysis, mechanical ventilation at 48 h was associated with subsequent HAP (OR = 2.19, 95% CI: 1.02–5.30, *p* = 0.06). Forced inclusion of tracheostomy did not materially alter core results.

**Conclusion:**

In severe TBI patients undergoing craniotomy, delayed enteral nutrition was independently associated with increased HAP risk, and HAP was associated with higher 30-day mortality. These findings highlight the potential importance of early enteral nutrition, but causal inference is limited by the observational design; prospective studies are needed.

## 1 Introduction

Severe traumatic brain injury (TBI) is the leading cause of trauma-related death and severe disability globally, imposing a heavy economic and medical burden on public health systems (1). For severe TBI patients with refractory intracranial hypertension or massive intracranial hematomas, craniotomy (such as decompressive craniectomy, hematoma evacuation, etc.) is often a critical life-saving intervention. However, these patients typically require transfer to a neuro-intensive care unit (NeuroICU) postoperatively, receiving prolonged invasive ventilation and invasive monitoring. Due to impaired consciousness, weakened cough reflexes, and continuously open artificial airways, the risk of postoperative nosocomial infections is extremely high. Among these, hospital-acquired pneumonia (HAP) is one of the most frequent and devastating complications in neurocritical care. Current data indicate that the incidence of pulmonary infections in severe TBI patients undergoing mechanical ventilation can be as high as 30 to 60% (2, 3); even in the broader neurosurgical population, the incidence of HAP following emergency craniotomy is significantly higher than that of elective surgeries (4, 5).

HAP is by no means merely a localized respiratory infection; rather, it is a critical link in a disastrous cascade affecting the neurological prognosis of TBI patients through “brain-lung crosstalk” (2). On one hand, severe brain injury induces a sympathetic storm, massive catecholamine release, and hyperactivation of the hypothalamic–pituitary–adrenal axis, driving the body into a “compensatory anti-inflammatory response syndrome”. This profound systemic immunosuppression severely undermines the lower respiratory tract’s ability to clear pathogenic bacteria (1, 3). On the other hand, the systemic inflammatory response syndrome, hypoxemia, and hypercapnia triggered by pulmonary infections can directly cause passive cerebral vasodilation, exacerbating blood–brain barrier disruption and cerebral edema, which subsequently leads to uncontrollable secondary intracranial hypertension and secondary cerebral hypoxia and ischemia (6, 7). Clinical observations have repeatedly confirmed that patients who develop postoperative pulmonary infections experience significantly prolonged ICU stays, and their short-term mortality rate can sharply increase to 20–50% (8). Therefore, the early identification and prevention of HAP are vital breakthroughs in improving outcomes following TBI surgery.

Although previous studies have extensively explored the risk factors for HAP after TBI surgery, many conclusions still focus on established independent predictors such as advanced age, low admission Glasgow Coma Scale (GCS) score, multiple trauma, and prolonged mechanical ventilation (9). In contrast, quantitative assessments of certain “highly modifiable factors” remain insufficient, particularly the timing of enteral nutrition initiation. The gastrointestinal tract is not only a digestive and absorptive organ but also the body’s largest immune center, maintaining systemic microecological balance (10, 11). The lack of early enteral nutrition leads to intestinal mucosal barrier atrophy and tight junction disruption, which triggers intestinal dysbiosis and the lymphatic-hematogenous translocation of pathogenic bacteria—this is precisely a critical mechanism for secondary infections in distant organs (such as the lungs) (12, 13). Based on this, both the American Society for Parenteral and Enteral Nutrition and the European Society for Clinical Nutrition and Metabolism strongly recommend initiating enteral nutrition within 24 to 48 h for critically ill patients with stable hemodynamics (14). However, in the real-world NeuroICU environment, because severe TBI patients often present with severe intracranial hypertension, delayed gastric emptying, or require continuous high-dose vasoactive drug support, clinicians habitually delay the initiation of enteral nutrition due to concerns about feeding intolerance and aspiration. To what extent this “delayed initiation (≥48 hours)” independently increases the risk of HAP in the post-craniotomy TBI population lacks large-sample, quantitatively adjusted evidence.

Given this, this study aims to systematically evaluate factors independently associated with HAP following craniotomy for TBI, with a particular focus on the timing of enteral nutrition initiation, and to assess the association between HAP and 30-day mortality. We hypothesized that earlier enteral nutrition and shorter invasive ventilation might be associated with a lower risk of HAP, but this study is not designed to establish causality.

## 2 Methods

### 2.1 Study design and ethical statement

This is a single-center, retrospective observational cohort study. The study protocol and reporting process strictly adhered to the Strengthening the Reporting of Observational Studies in Epidemiology (STROBE) statement and the Declaration of Helsinki. The study protocol was reviewed and approved by the Ethics Committee of Xinxiang Central Hospital (Approval No.: 2020-122). Given the retrospective and non-interventional nature of the study, patient information was de-identified and anonymized, and the ethics committee formally waived the requirement for patient informed consent.

### 2.2 Study population and grouping

Adult patients (≥18 years) who were admitted for TBI and underwent craniotomy (including hematoma evacuation, decompressive craniectomy, and combined surgeries) between 2023 and 2025 were consecutively included.

*Inclusion criteria*: (1) confirmed diagnosis of severe TBI requiring craniotomy; (2) postoperative survival > 48 h.

*Exclusion criteria*: (1) presence of pulmonary infection or confirmed severe infection at other sites upon admission or within 48 h prior to surgery; (2) > 20% missing data for key study variables (e.g., GCS score, ISS score, timing of enteral nutrition initiation).

Patients were divided into an HAP group and a non-HAP group based on whether they developed HAP during the postoperative period.

HAP was diagnosed according to the Centers for Disease Control and Prevention (CDC)/National Healthcare Safety Network (NHSN) criteria: new or progressive pulmonary infiltrates on chest imaging occurring >48 h after surgery, accompanied by at least two of the following: fever (>38.3 °C), leukocytosis or leukopenia, purulent sputum, or positive microbiological culture. All suspected cases were independently reviewed by two neurocritical care physicians; disagreements were resolved by a radiologist. For patients meeting clinical criteria, respiratory specimens (sputum or endotracheal aspirate) were obtained for Gram stain and semi-quantitative culture. In this study, mechanically ventilated patients who developed pneumonia after 48 h also met the definition of ventilator-associated pneumonia (VAP); however, to maintain consistency, the term HAP was used throughout the manuscript. The time from surgery to HAP diagnosis was recorded.

### 2.3 Data collection

Patient data were structurally extracted through the electronic medical record system: (1) Demographic and baseline characteristics: Age, gender, body mass index (BMI), smoking status, and underlying diseases (hypertension, diabetes); (2) Injury and surgical characteristics: Admission GCS score, Injury Severity Score (ISS), and surgery type; (3) Postoperative management and laboratory indicators: Days of mechanical ventilation, tracheostomy status, postoperative serum albumin levels, and the initiation timing of enteral nutrition (EN). In this study, the timing of EN initiation was dichotomized into early initiation (< 48 h) and delayed initiation (≥ 48 h).

### 2.4 Outcome measures

*Primary outcome*: incidence of postoperative HAP.

*Secondary outcomes*: ICU length of stay and 30-day mortality.

### 2.5 Statistical analysis

Normally distributed continuous variables are presented as mean ± SD and compared using the independent samples t-test; non-normally distributed continuous variables are presented as median and interquartile range (IQR) and compared using the Mann–Whitney U test. Categorical variables are expressed as frequencies and percentages (*n*, %) and compared between groups using the chi-square test or Fisher’s exact test.

Variables with *p* < 0.10 in the univariate analysis and those considered clinically important were included in the multivariable logistic regression model to identify factors associated with HAP. Multicollinearity was assessed using the variance inflation factor (VIF < 5 indicating no significant collinearity), and model calibration was evaluated using the Hosmer–Lemeshow test.

To avoid reverse causation, the duration of mechanical ventilation in the primary HAP model was defined as follows: for patients who developed HAP, it was calculated from the start of postoperative mechanical ventilation to the time of HAP diagnosis; for patients who did not develop HAP, it was calculated from the start of postoperative mechanical ventilation to the end of mechanical ventilation. As a landmark sensitivity analysis to address the potential bias from different exposure windows for mechanical ventilation, we used postoperative 48 h as the landmark time point. Patients who developed HAP or died before 48 h were excluded from this analysis; in practice, all included patients survived >48 h and HAP was defined as occurring >48 h after surgery, so no additional exclusions were needed. Mechanical ventilation status at 48 h was treated as a binary exposure, and its association with subsequent HAP was assessed using multivariable logistic regression adjusting for the same covariates as the primary model, including delayed enteral nutrition, age, admission GCS score, and serum albumin. Follow-up started at 48 h and continued until HAP, death, discharge, or 30 days.

For the 30-day mortality outcome, in addition to age, admission GCS, and ISS, the following covariates were included to reduce confounding: mechanical ventilation duration, serum albumin, enteral nutrition initiation timing, tracheostomy, hypertension, and diabetes. In the time-dependent Cox model, mechanical ventilation was treated as a time-dependent exposure according to the actual start and stop times, without truncation at HAP onset, because the time-dependent structure already accounts for temporal ordering.

Receiver operating characteristic (ROC) curves were plotted, and the area under the curve (AUC) was calculated to evaluate model discrimination. A two-sided *p* < 0.05 was considered statistically significant. All statistical analyses were performed using R software (version 4.2.0).

Detailed data on hemodynamic instability, vasopressor dosages, feeding intolerance, aspiration events, and intracranial pressure management were not available in the retrospective database and therefore could not be included in the multivariable models.

## 3 Results

### 3.1 Patient baseline characteristics and HAP incidence

A total of 600 patients who underwent craniotomy for severe TBI were included in the analysis. Among them, 69 patients (11.5%) developed hospital-acquired pneumonia (HAP) postoperatively. As shown in Table 1, univariate analysis indicated that compared with patients who did not develop HAP, those in the HAP group were older (62.41 ± 15.27 vs. 55.02 ± 14.84 years, *p* < 0.001) and had a lower admission GCS score (6.42 ± 2.93 vs. 8.01 ± 3.48, *p* < 0.001). With respect to treatment and nutritional management, the total mechanical ventilation duration in the HAP group was significantly longer (7.58 ± 6.65 vs. 5.30 ± 4.22 days, *p* < 0.001), serum albumin levels were lower (33.20 ± 5.20 vs. 35.07 ± 4.94 g/L, *p* = 0.003), and the proportion of delayed enteral nutrition initiation (≥ 48 h) was significantly higher (68.1% vs. 37.1%, *p* < 0.001). There was no statistically significant difference in smoking status between the two groups (*p* = 0.568). Regarding healthcare resource utilization (Figure 1), the ICU length of stay was significantly longer in the HAP group than in the non-HAP group (median 14 days, IQR 11–18 vs. median 7 days, IQR 5–9; *p* < 0.001). Among the 69 patients with HAP, the median time from surgery to HAP diagnosis was 10 days (IQR, 5.5–10.75). Radiological assessment was performed jointly by ICU physicians and radiologists. Microbiological culture results were not consistently recorded in the retrospective database; therefore, the pathogen spectrum and microbiological positivity rates could not be reported.

**Table 1:** Baseline characteristics of patients with and without hospital-acquired pneumonia after TBI surgery.

Variable	Total (*n* = 600)	Non-HAP group (*n* = 531)	HAP group (*n* = 69)	*p*-value
Demographics				
Age (years), mean ± SD	55.87 ± 15.10	55.02 ± 14.84	62.41 ± 15.27	< 0.001
Male sex, n (%)	400 (66.7)	354 (66.7)	46 (66.7)	1.000
BMI, mean ± SD	23.90 ± 3.01	23.93 ± 2.99	23.65 ± 3.10	0.465
Smoking status, *n* (%)				0.568
Never smoked	290 (48.3)	256 (48.2)	34 (49.3)	
Former smoker	123 (20.5)	112 (21.1)	11 (15.9)	
Current smoker	187 (31.2)	163 (30.7)	24 (34.8)	
Underlying diseases				
Hypertension, *n* (%)	210 (35.0)	184 (34.7)	26 (37.7)	0.717
Diabetes, *n* (%)	132 (22.0)	118 (22.2)	14 (20.3)	0.834
Injury and surgical characteristics				
Admission GCS score, mean ± SD	7.83 ± 3.46	8.01 ± 3.48	6.42 ± 2.93	< 0.001
ISS score, mean ± SD	24.05 ± 7.92	24.15 ± 7.98	23.25 ± 7.43	0.373
Surgery type, *n* (%)				0.397
Hematoma evacuation	194 (32.3)	172 (32.4)	22 (31.9)	
Decompressive craniectomy	218 (36.3)	190 (35.8)	28 (40.6)	
Combined surgery	188 (31.3)	169 (31.8)	19 (27.5)	
Postoperative management and laboratory indicators				
Mechanical ventilation, days, mean ± SD	5.56 ± 4.63	5.30 ± 4.22	7.58 ± 6.65	< 0.001
Tracheostomy, *n* (%)	110 (18.3)	94 (17.7)	16 (23.2)	0.346
Enteral nutrition initiation ≥ 48 h, *n* (%)	244 (40.7)	197 (37.1)	47 (68.1)	< 0.001
Serum albumin, g/L, mean ± SD	34.85 ± 5.00	35.07 ± 4.94	33.20 ± 5.20	0.003

Continuous variables are presented as mean ± SD and were compared using the independent samples t-test. Categorical variables are presented as frequencies and percentages and were compared using the chi-square test or Fisher’s exact test, as appropriate. HAP, hospital-acquired pneumonia; BMI, body mass index; GCS, Glasgow Coma Scale; ISS, Injury Severity Score.

**Figure 1:**
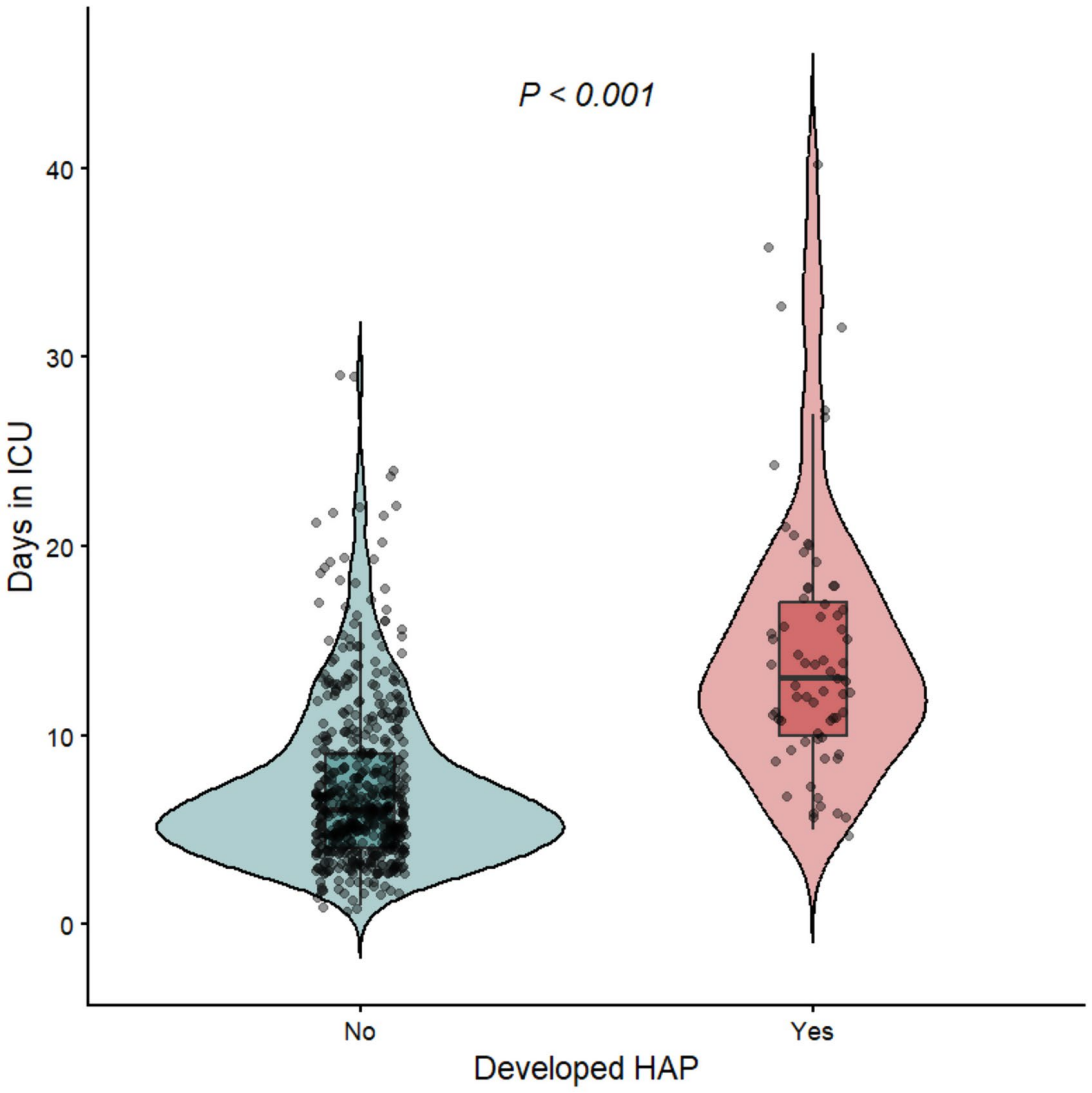
Comparison of ICU length of stay between HAP and non-HAP groups. The violin plot combined with a scatter plot illustrates the distribution of ICU length of stay in the two groups. The embedded box plots indicate the median and interquartile range. Groups were compared using the Mann–Whitney U test; the difference was statistically significant (*p* < 0.001).

### 3.2 Analysis of independent factors associated with developing HAP post-TBI surgery

To further control for confounding factors, variables with *p* < 0.10 in the univariate analysis and those considered clinically important were included in a multivariable logistic regression model. Multicollinearity was assessed using the variance inflation factor (VIF), with all VIF values less than 2, and the model fit was good (Hosmer–Lemeshow test *p* > 0.05). In the primary model, which used pre-HAP ventilation duration for HAP patients, each additional day of mechanical ventilation remained associated with an increased risk of HAP (OR = 1.11, 95% CI: 1.05–1.17, *p* < 0.001). Delayed initiation of enteral nutrition (≥48 h) was independently associated with an increased risk of HAP (OR = 4.46, 95% CI: 2.52–8.19, *p* < 0.001). Additionally, advanced age and longer mechanical ventilation duration were also associated with a higher risk of HAP. Conversely, a higher admission GCS score and higher serum albumin levels were associated with a lower risk of HAP. This multivariable prediction model demonstrated good discrimination (Figure 2), with an area under the receiver operating characteristic curve (AUC) of 0.795 (95% CI: 0.744–0.848) (see Tables 2, 3).

**Figure 2:**
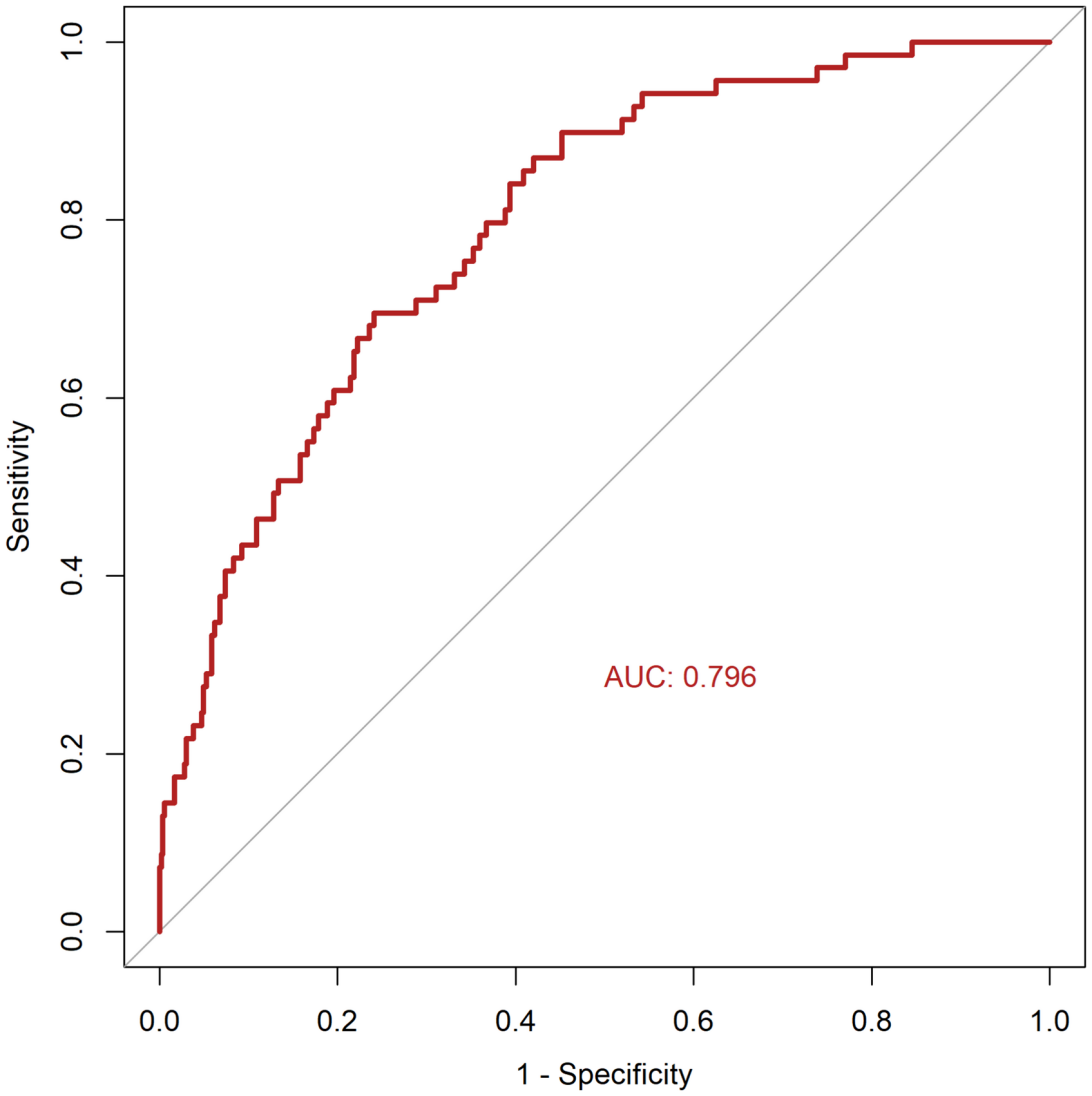
Receiver operating characteristic curve for the multivariable model predicting hospital-acquired pneumonia after TBI surgery. The model demonstrated good discrimination, with an area under the curve of 0.795 (95% CI: 0.744–0.848, DeLong method) AUC, area under the curve; CI, confidence interval; TBI, traumatic brain injury.

**Table 2:** Multivariable logistic regression analysis of factors associated with HAP following TBI surgery.

Variable	Adjusted OR	95% CI	*p-*value
Age (per 1-year increase)	1.03	1.02–1.05	<0.001
Admission GCS (per 1-point increase)	0.86	0.78–0.94	0.001
Mechanical ventilation (per 1-day increase, before HAP onset for HAP patients)	1.11	1.05–1.17	<0.001
Enteral nutrition ≥48 h (vs. <48 h)	4.46	2.52–8.19	<0.001
Serum albumin (per 1 g/L)	0.91	0.86–0.96	0.001

The primary model used mechanical ventilation duration truncated at the time of HAP diagnosis for HAP patients; total mechanical ventilation duration was used for non-HAP patients. OR, adjusted odds ratio; CI, confidence interval.

**Table 3:** Time-dependent Cox regression analysis of HAP and clinical covariates associated with 30-day mortality after TBI surgery.

Variable	HR	95% CI	*p*-value
Time-dependent exposures			
HAP (exposed after diagnosis)	4.31	1.52–12.25	0.006
Mechanical ventilation (exposed during ventilation)	0.85	0.81–0.89	<0.001
Fixed covariates			
Age (per year)	1.04	1.03–1.05	<0.001
Admission GCS (per point)	0.88	0.82–0.93	<0.001
ISS score	1.00	0.98–1.02	0.984
Serum albumin (per g/L)	1.02	0.99–1.06	0.261
Enteral nutrition ≥48 h (vs. <48 h)	0.89	0.63–1.27	0.527
Hypertension (vs. none)	1.47	1.03–2.10	0.034
Diabetes (vs. none)	0.60	0.38–0.96	0.031
Male sex (vs. female)	1.58	1.05–2.37	0.027
Smoking (former vs. never)	1.17	0.69–1.98	0.570
Smoking (current vs. never)	1.15	0.78–1.69	0.477
Surgery type (decompressive craniectomy vs. hematoma evacuation)	0.87	0.57–1.33	0.530
Surgery type (both vs. hematoma evacuation)	0.94	0.61–1.42	0.757

HR, hazard ratio; CI, confidence interval. The model adjusted for all variables listed; HAP and mechanical ventilation were included as time-dependent covariates, while the remaining covariates were fixed. Model concordance (C-statistic) = 0.804. As a sensitivity analysis, the extended multivariable logistic regression model showed that HAP was associated with 30-day mortality (OR = 6.23, 95% CI: 3.29–12.14), consistent in direction with the time-dependent Cox model. When HAP was treated as a fixed covariate in a conventional Cox model, the HR was 0.51 (95% CI: 0.33–0.81), indicating immortal time bias; this is further discussed in the Discussion section.

### 3.3 Independent impact of HAP on 30-day mortality

To clarify the impact of HAP on adverse early clinical outcomes, a time-dependent Cox proportional hazards model was constructed with 30-day mortality as the dependent variable. In this model, HAP was treated as a time-dependent exposure, and mechanical ventilation was also treated as a time-dependent exposure according to the actual start and stop times. After adjusting for age, admission GCS score, ISS score, and other fixed covariates, HAP was significantly associated with an increased risk of 30-day mortality (HR = 4.31, 95% CI: 1.52–12.25, *p* = 0.006). Mechanical ventilation as a time-dependent exposure showed an HR of 0.85 (95% CI: 0.81–0.89). Other factors associated with mortality included age (HR = 1.04, 95% CI: 1.03–1.05), admission GCS score (HR = 0.88, 95% CI: 0.82–0.93), hypertension (HR = 1.47, 95% CI: 1.03–2.10), diabetes (HR = 0.60, 95% CI: 0.38–0.96), and male sex (HR = 1.58, 95% CI: 1.05–2.37). As a sensitivity analysis, the extended multivariable logistic regression model showed that HAP was associated with 30-day mortality (OR = 6.23, 95% CI: 3.29–12.14), consistent in direction with the time-dependent Cox model. By contrast, when HAP was treated as a fixed covariate in a conventional Cox regression, the HR was 0.51 (95% CI: 0.33–0.81), indicating immortal time bias; the time-dependent Cox model corrected this bias. Similarly, the multivariable logistic regression model (OR = 6.23) should not be interpreted as a direct causal effect, because it does not account for the timing of HAP onset and is subject to immortal time bias (see Figures 3, 4).

**Figure 3:**
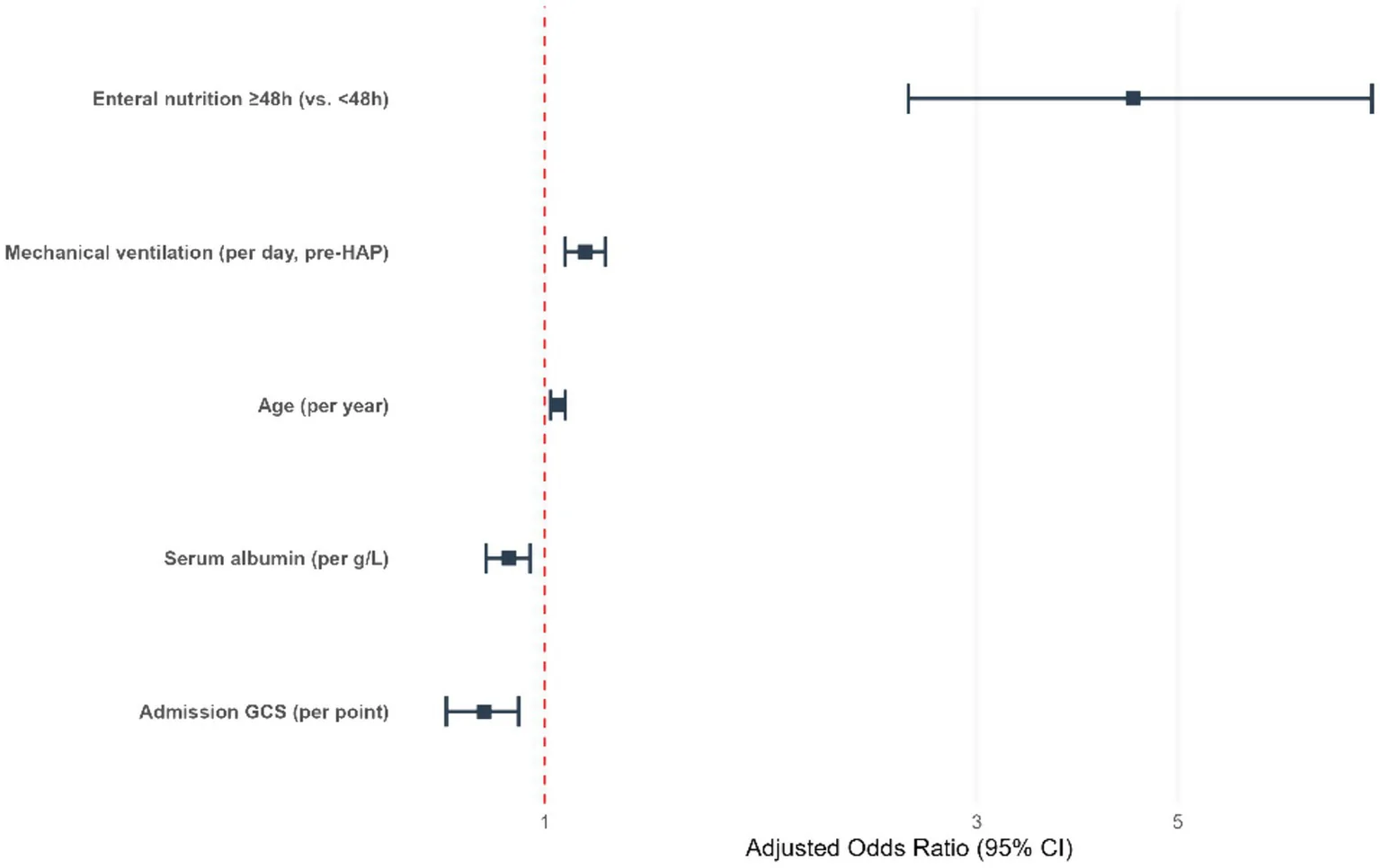
Forest plot of factors associated with hospital-acquired pneumonia after TBI surgery. Squares represent adjusted odds ratios calculated from the multivariable logistic regression model; horizontal lines represent 95% confidence intervals. The red dashed line indicates an odds ratio of 1. Mechanical ventilation duration was truncated at HAP onset for HAP patients. OR, odds ratio; CI, confidence interval; HAP, hospital-acquired pneumonia; TBI, traumatic brain injury.

**Figure 4:**
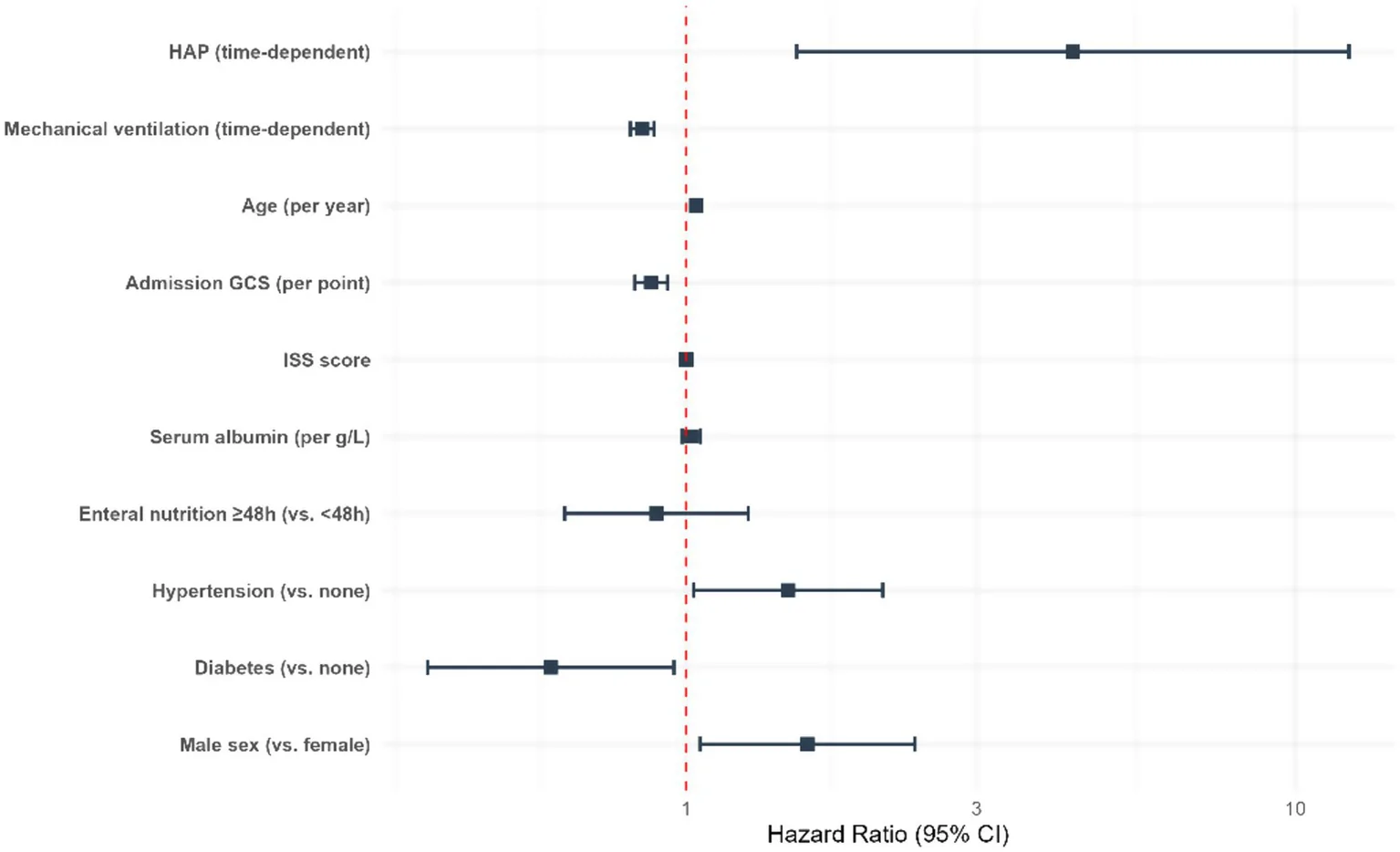
Forest plot showing results of the time-dependent Cox regression model for 30-day mortality. Squares represent adjusted hazard ratios (HRs) derived from the time-dependent Cox model; horizontal lines represent 95% confidence intervals (CIs). The red dashed line indicates an HR of 1. HAP and mechanical ventilation were treated as time-dependent exposures; the remaining covariates were fixed. HR, hazard ratio; CI, confidence interval; HAP, hospital-acquired pneumonia.

### 3.4 Sensitivity analysis

To assess the robustness of the findings, two additional analyses were performed.

First, because the primary model used mechanical ventilation duration truncated at the time of HAP diagnosis for HAP patients, we compared it with the original model using total ventilation duration. The results were nearly identical: the OR for mechanical ventilation per additional day was 1.11 (95% CI: 1.05–1.17) in the primary model and 1.11 (95% CI: 1.05–1.17) in the original model, and the ORs of other variables changed by less than 5% (Supplementary Table S1). This indicates that the choice of ventilation time definition had minimal impact on the results and that reverse causation is unlikely to substantially affect the association.

Second, we forced tracheostomy into the multivariable model. In this analysis, tracheostomy was significantly associated with a lower risk of HAP (OR = 0.39, 95% CI: 0.15–0.95, *p* = 0.045), while the associations of the main variables—including delayed enteral nutrition, age, admission GCS score, and serum albumin—remained materially unchanged (Supplementary Table S3). Third, a landmark analysis using mechanical ventilation status at 48 h postoperatively showed that mechanically ventilated patients had a higher risk of subsequent HAP (OR = 2.19, 95% CI: 1.02–5.30, *p* = 0.06), while delayed enteral nutrition remained independently associated (OR = 3.68, 95% CI: 2.13–6.56, *p* < 0.001) (Supplementary Table S4).

However, these sensitivity analyses could not fully account for unmeasured confounding by indication, as detailed in the Discussion.

## 4 Discussion

Using a retrospective cohort design, this study systematically quantified the clinical burden, factors independently associated with HAP, and the impact of HAP on short-term prognosis in patients undergoing craniotomy for severe traumatic brain injury (TBI). Among the 600 surgical patients with severe TBI included, the incidence of postoperative HAP was 11.5%. Although this figure is at the lower end of the reported pulmonary infection rates following neurosurgery (10–60%) (2–4), this is primarily because our study excluded patients who already had infections upon admission and restricted the inclusion criteria to those surviving beyond 48 h postoperatively, thus more purely reflecting the true burden of perioperative iatrogenic HAP. More importantly, in the time-dependent Cox model, HAP was associated with a significantly higher risk of 30-day mortality (HR = 4.31, 95% CI: 1.52–12.25). This finding suggests that HAP is not merely an epiphenomenon, but an independent factor associated with higher short-term mortality.

The association between HAP and higher post-TBI mortality may be related to the “brain–lung crosstalk” that has attracted considerable attention in recent years; however, the present study did not directly measure relevant biological markers, so this mechanism remains speculative (1, 2). Severe TBI not only directly damages the central nervous system but also induces a sympathetic storm that causes the massive peripheral release of catecholamines, leading to an acute immunosuppressive state. This subsequently impairs the respiratory mucosal barrier and significantly increases susceptibility to opportunistic pathogens (3). Once HAP occurs, a large accumulation of pro-inflammatory cytokines in the alveoli may cross the damaged blood–brain barrier into the central nervous system, activating microglia and triggering neurotoxic inflammation (6). Concurrently, systemic hypoxemia and hypercapnia induced by the pulmonary infection can directly cause passive cerebral vasodilation, leading to an intractable elevation in intracranial pressure and accelerating brain herniation (7). This bidirectional vicious cycle between the brain and lungs may be one of the potential mechanisms linking pulmonary infection to poor prognosis; however, because blood–brain barrier permeability, microglial activation, and inflammatory cytokine levels were not measured in this study, this pathway could not be confirmed.

In exploring the specific factors associated with HAP, this study reaffirmed that advanced age and a lower admission GCS score are non-modifiable independent factors, highly consistent with multiple studies on postoperative pulmonary complications in neurosurgery (4). A low GCS score directly reflects severe consciousness impairment and profound suppression of swallowing function, making micro-aspiration of oropharyngeal secretions and gastroesophageal reflux a continuous problem. Among potentially modifiable treatment factors, each additional day of mechanical ventilation was associated with an 11% higher risk of HAP. A landmark analysis at 48 h showed a consistent association, though the effect was marginally significant in this binary specification. Tracheal intubation completely destroys the natural physiological barrier of the respiratory tract, and bacterial biofilms formed on the surface of the catheter provide a direct pathway for the colonization of multidrug-resistant bacteria (9). Furthermore, postoperative hypoalbuminemia was also associated with an increased risk of infection. This may reflect not only the intensity of the systemic inflammatory response accompanying the decline in negative acute-phase proteins, but also a reduction in colloid osmotic pressure, which can further exacerbate pulmonary interstitial edema and weaken local alveolar immune defenses (7).

The most clinically relevant finding of this study is that delayed initiation of enteral nutrition (≥48 h) was independently associated with an increased risk of HAP after TBI surgery (OR = 4.46, 95% CI: 2.52–8.19). As the body’s largest peripheral immune organ, the gut-associated lymphoid tissue houses approximately 70% of the body’s immune cells (10). If early enteral nutritional stimulation is absent following TBI, the intestinal mucosal mechanical barrier may rapidly atrophy, potentially leading to intestinal dysbiosis and pathogen translocation—a proposed pathway for triggering distal “gut-derived pulmonary infections” (12). Both the American Society for Parenteral and Enteral Nutrition and the European Society for Clinical Nutrition and Metabolism guidelines strongly recommend initiating enteral nutrition within 24–48 h for critically ill patients with stable hemodynamics (13). A propensity score-matched analysis based on a nationwide inpatient database in Japan also reported that early enteral nutrition was associated with a lower incidence of hospital-acquired pneumonia in patients with severe TBI (12). These findings are consistent with current guideline recommendations and suggest that early enteral nutrition may play a role beyond nutritional support, possibly by helping to maintain intestinal barrier function and immune homeostasis. However, the present study did not directly assess gut barrier integrity, microbiota composition, or pathogen translocation, so this interpretation remains speculative.

To test the robustness of our core findings, we also performed a sensitivity analysis forcing the inclusion of tracheostomy. The results showed that, under the premise of simultaneously adjusting for mechanical ventilation duration and enteral nutrition initiation timing, tracheostomy did not show a significant additional effect, and the OR values of major risk factors remained highly stable, further consolidating the reliability of our study conclusions.

Although this study constructed a rigorously adjusted model, several limitations exist. First, as a retrospective cohort study, it cannot completely rule out biases introduced by unrecorded residual confounders (5). Second, because this was a retrospective study, the precise temporal sequence between certain variables and HAP could not be fully determined. For example, total mechanical ventilation duration may have been prolonged by HAP itself, which could have exaggerated its association with HAP. To evaluate this potential bias, we truncated the mechanical ventilation time at HAP diagnosis for HAP patients and re-analyzed the data; the OR remained nearly unchanged, suggesting that reverse causation had a limited impact on the primary findings. However, delayed enteral nutrition and HAP may still be affected by confounding by indication—namely, more severely ill patients were both more likely to have delayed feeding and more likely to develop HAP. Although we adjusted for GCS and ISS scores and other severity indicators, unmeasured confounding cannot be fully excluded. The most important limitation is that we could not adjust for APACHE II/SOFA scores, shock status, lactate levels, vasoactive drug dosages, postoperative ICP control, sedation depth, gastric residual volume, vomiting/aspiration, pulmonary contusion/thoracic trauma, or antibiotic exposure. These variables may influence both the timing of enteral nutrition initiation and the risk of HAP. Therefore, delayed enteral nutrition may partly serve as a surrogate marker of critical illness rather than a directly modifiable causal factor. Accordingly, the observed association should be interpreted with caution.

By supplementing data on death dates and last follow-up dates, we used a time-dependent Cox model with HAP as a time-dependent exposure, thereby effectively correcting for immortal time bias. The results showed that HAP was associated with an approximately four-fold higher risk of 30-day mortality, consistent with the extended logistic regression model. The conventional logistic regression estimate should therefore be interpreted cautiously and is not considered the primary result. This suggests that HAP was not simply identified because patients survived longer; rather, it was independently associated with poor prognosis. Notably, in a conventional Cox regression treating HAP as a fixed covariate, the HR was 0.51, which is opposite in direction and clearly demonstrates the bias caused by ignoring time dependency. Our time-dependent analysis provides more reliable evidence for this association. However, the HR for mechanical ventilation as a time-dependent exposure was less than 1, which may reflect closer monitoring and support during mechanical ventilation; this result should be interpreted cautiously.

Another limitation is that the retrospective database did not consistently record microbiological culture results, so the pathogen spectrum and microbiological positivity rates for HAP could not be reported. In addition, the use of empirical antibiotics before culture collection may have reduced culture positivity. The relatively low HAP incidence (11.5%) may be partly explained by the exclusion of patients with pre-existing infection at admission and those who died within 48 h after surgery, as well as the requirement of survival >48 h, which excluded very early postoperative deaths. This definition likely captured a more selected cohort of patients who survived long enough to develop HAP, thereby reflecting the true burden of perioperative nosocomial pneumonia. Furthermore, because culture data were incomplete and some patients may have received empirical antibiotics before specimen collection, the microbiological positivity rate of HAP may have been underestimated.

In addition, the association between delayed enteral nutrition and HAP observed in this study is particularly susceptible to confounding by indication. Patients who could not receive early enteral nutrition were often more severely ill, with potential hemodynamic instability, higher vasopressor requirements, feeding intolerance, elevated intracranial pressure, or other contraindications to early feeding. These clinical factors may themselves increase the risk of HAP. Although we adjusted for several severity indicators, including the GCS and ISS, the retrospective database did not record detailed hemodynamic parameters, vasopressor doses, gastric residual volumes, aspiration events, or intracranial pressure management. Therefore, delayed enteral nutrition may partly serve as a surrogate marker of critical illness rather than being a directly modifiable causal factor. Accordingly, the observed association should be interpreted with caution, and future prospective studies with detailed physiological data are needed to address this potential bias.

Although we adjusted for GCS and ISS scores in the prognostic model, biases caused by forced feeding delays due to extreme critical illness are difficult to eliminate entirely. Additionally, limited by data availability, we were unable to include dynamic neuromonitoring indicators, such as the pupillary light reflex, in the analysis (15). Future prospective randomized controlled trials, or quasi-experimental designs such as instrumental variable analysis and propensity score matching, are urgently needed to further verify the association between early enteral nutrition and HAP reduction, thereby providing higher-level evidence-based medical support for bundled neurocritical care management.

## Data Availability

The raw data supporting the conclusions of this article will be made available by the authors, without undue reservation.
