# Supplementary material for: Association of delayed enteral nutrition with hospital-acquired pneumonia and 30-day mortality following craniotomy for severe traumatic brain injury: a retrospective cohort study
Source: Front Nutr. 2026 Oct 1;13:1911672. doi: 10.3389/fnut.2026.1911672 (PMC13668601; doi:10.3389/fnut.2026.1911672)
Supplement: Supplementary file 1 [file Table_1.docx]

**Table S1. Comparison of primary model (pre-HAP ventilation duration) and original model (total ventilation duration) for HAP**

| Variable | Primary model OR (95% CI) | Original model OR (95% CI) |
| --- | --- | --- |
| Age | 1.03 (1.02–1.05) | 1.03 (1.02–1.05) |
| Admission GCS | 0.86 (0.78–0.94) | 0.86 (0.78–0.94) |
| Mechanical ventilation | 1.11 (1.05–1.17) | 1.11 (1.05–1.17) |
| Enteral nutrition ≥48h | 4.46 (2.52–8.19) | 4.46 (2.52–8.19) |
| Serum albumin | 0.91 (0.86–0.96) | 0.91 (0.86–0.96) |

**Table S2. Sensitivity analysis using extended multivariable logistic regression for 30-day mortality after TBI craniotomy**

| Variable | Adjusted OR | 95% CI | *P-*value |
| --- | --- | --- | --- |
| HAP (vs. no HAP) | 6.23 | 3.29–12.14 | <0.001 |
| Age (per year) | 1.06 | 1.05–1.08 | <0.001 |
| Admission GCS (per point) | 0.82 | 0.76–0.89 | <0.001 |
| ISS score | 0.98 | 0.96–1.01 | 0.263 |
| Mechanical ventilation (per day) | 1.05 | 0.99–1.13 | 0.130 |
| Serum albumin (per g/L) | 1.03 | 0.99–1.08 | 0.195 |
| Enteral nutrition ≥48 h (vs. <48 h) | 1.26 | 0.79–2.01 | 0.324 |
| Tracheostomy (vs. none) | 0.69 | 0.31–1.51 | 0.363 |
| Hypertension (vs. none) | 1.72 | 1.09–2.72 | 0.021 |
| Diabetes (vs. none) | 0.64 | 0.35–1.14 | 0.144 |
| Note: The model adjusted for all variables listed. HAP: hospital-acquired pneumonia; GCS: Glasgow Coma Scale; ISS: Injury Severity Score. Model discrimination: AUC=0.826; Hosmer–Lemeshow test P>0.05. This logistic model served as a sensitivity analysis to the time-dependent Cox regression. The direction of the association for HAP was consistent with that of the time-dependent Cox model (HR=4.31), supporting robustness. | | | |

**Table S3. Sensitivity analysis forcing tracheostomy into the multivariable model for HAP**

| Variable | Adjusted OR | 95% CI | *P-*value |
| --- | --- | --- | --- |
| Age (per year) | 1.03 | 1.01–1.05 | <0.001 |
| Admission GCS (per point) | 0.85 | 0.78–0.93 | 0.001 |
| Mechanical ventilation (per day) | 1.17 | 1.09–1.26 | <0.001 |
| Enteral nutrition ≥48 h (vs. <48 h) | 4.66 | 2.61–8.63 | <0.001 |
| Serum albumin (per g/L) | 0.91 | 0.86–0.96 | 0.001 |
| Tracheostomy (vs. none) | 0.39 | 0.15–0.95 | 0.045 |
| Note: The model included all variables listed plus age, GCS, mechanical ventilation days, enteral nutrition timing, albumin, and tracheostomy. OR: adjusted odds ratio; CI: confidence interval. | | | |

**Table S4. Landmark sensitivity analysis using mechanical ventilation status at 48 hours postoperatively**

| Variable | Adjusted OR | 95% CI | *P* value |
| --- | --- | --- | --- |
| Age (per year) | 1.04 | 1.02–1.06 | <0.001 |
| Admission GCS (per point) | 0.86 | 0.78–0.93 | 0.001 |
| Mechanical ventilation at 48 h (vs. not) | 2.19 | 1.02–5.30 | 0.060 |
| Enteral nutrition ≥48 h (vs. <48 h) | 3.68 | 2.13–6.56 | <0.001 |
| Serum albumin (per g/L) | 0.91 | 0.86–0.96 | 0.001 |
| **Note:** Landmark analysis was performed at 48 hours postoperatively. Mechanical ventilation status was defined as receiving invasive mechanical ventilation at exactly 48 hours after surgery. OR, adjusted odds ratio; CI, confidence interval. | | | |
